# Evolution of a subtilisin-like protease gene family in the grass endophytic fungus *Epichloë festucae*

**DOI:** 10.1186/1471-2148-9-168

**Published:** 2009-07-19

**Authors:** Michelle K Bryant, Christopher L Schardl, Uljana Hesse, Barry Scott

**Affiliations:** 1 Institute of Molecular Biosciences, Massey University, Private Bag 11222, Palmerston North, New Zealand; 2 Department of Plant Pathology, University of Kentucky, Lexington, KY 40546-0312, USA

## Abstract

**Background:**

Subtilisin-like proteases (SLPs) form a superfamily of enzymes that act to degrade protein substrates. In fungi, SLPs can play either a general nutritive role, or may play specific roles in cell metabolism, or as pathogenicity or virulence factors.

**Results:**

Fifteen different genes encoding SLPs were identified in the genome of the grass endophytic fungus *Epichloë festucae*. Phylogenetic analysis indicated that these SLPs belong to four different subtilisin families: proteinase K, kexin, pyrolysin and subtilisin. The pattern of intron loss and gain is consistent with this phylogeny. *E. festucae *is exceptional in that it contains two kexin-like genes. Phylogenetic analysis in Hypocreales fungi revealed an extensive history of gene loss and duplication.

**Conclusion:**

This study provides new insights into the evolution of the SLP superfamily in filamentous fungi.

## Background

Proteases catalyse the cleavage of polypeptides to oligopeptides or amino acids. In fungi, aspartic, cysteine, metallo-, serine and threonine proteases, as well as uncharacterised classes of proteases, have been identified, which have been classified according to the amino acid residues required for catalytic activity [1]. The serine proteases represent the most well known class, with two major superfamilies: subtilisin-like proteases (SLPs) and the trypsins. Both superfamilies use the same catalytic triad (Asp-His-Ser), which is thought to have evolved through convergent evolution [2].

Subtilisin-like proteases (SLPs) are ubiquitous in prokaryotes and eukaryotes. Six families of SLPs have been identified [3]: subtilisins, proteinase K-type, thermitases, kexins, lantibiotic peptidases and pyrolysins. Phylogenomic analyses suggest three families of subtilisin-like proteases are present in fungi [4]. The first family, known as proteinase K-type, was first identified in fungi and named for its similarity to the widely known *Tritirachium album *proteinase K [5]. These proteases are generally characterised by the presence of subtilisin N-terminal domain containing the propeptide, which is thought to act as an intramolecular chaperone to assist protein folding as well as inhibit enzyme activity [6,7], and a catalytic peptidase S8 domain. Phylogenetic analyses suggest subfamilies 1 and 2 of this family contain secreted proteases, whereas subfamily 3 contains intracellular proteases localised to the vacuole [4]. The secreted proteases are thought to generally play a nutritive role [8], but the vacuolar proteases appear to play a specialised role in the breakdown of autophagic bodies in the vacuole during autophagy, allowing recycling of macromolecules during nutrient starvation [9,10].

The second family of SLPs identified in fungi is the kexins. Kexins have two major domains: a peptidase S8 catalytic domain, and a proprotein convertase domain. Kexin-type enzymes, first identified in the yeast *Saccharomyces cerevisiae *[11], play an important role in post-translational modification in eukaryotes. Secreted proteins in eukaryotes are often synthesized as preproproteins, which undergo two proteolytic processing events to become mature proteins. The prepeptide is normally removed by a signal peptidase in the endoplasmic reticulum [12]. The resulting proprotein is then transferred to the Golgi, where kexin-like enzymes cleave the propeptide to give the mature protein.

The third class was described as class I, or members of the subtilisin family [4]. Members of this family in fungi usually have inserts in the catalytic domain, and long carboxyl-terminal extensions, which are both characteristic of a family described as pyrolysins [3]. However, the pyrolysin family appears to be heterogeneous, with many different accessory domains. The class I subtilisins generally contain a protease-associated (PA) domain inserted into the catalytic domain [13], along with a DUF1034 domain (this study), which has an unknown function.

The subtilisin superfamily is an interesting case study for the evolution of multigene families. Gene duplication (and subsequent divergence) along with gene loss are important contributors to gene family evolution [14,15]. Gene loss can occur through either loss of gene function due to deleterious mutations or through complete deletion of the gene. There is evidence of extensive gene duplication and loss within the SLP family in fungal lineages, which has been correlated with differences in fungal lifestyles [4].

In this study, we examined the evolution of the SLP gene family from the endophytic fungus, *Epichloë festucae*. This fungus forms a mutually beneficial association with its host grass. We were interested in the gene family in this organism because of its plant symbiotic lifestyle and close taxonomic relationship to the insect pathogen *Metarhizium anisopliae *(both Clavicipitaceae), where SLPs are important as virulence factors. The availability of other fungal genomes, especially those from *Fusarium*, *Nectria *and *Trichoderma *spp. also allows comparisons of SLP family evolution in fungi through gene duplication, loss and divergence.

## Methods

### Bacterial strains and plasmids

*E. coli *strains were grown on LB agar plates, supplemented with ampicillin (100 μg/mL) where necessary.

### Fungal strains and growth conditions

Cultures of *Neotyphodium lolii *strain Lp19 [16] and *E. festucae *strain Fl1 (ex cultivar SR3000) were grown and maintained as described previously [17,18].

### Molecular biology techniques

Fungal genomic DNA was isolated from freeze-dried mycelia using previously described methods [19,20]. Plasmid DNA was isolated and purified by alkaline lysis using either the Bio-Rad (Hercules, CA 94547, USA) Quantum plasmid miniprep or midiprep kits or the Roche (Roche Diagnostics N.Z., Ltd., Auckland, New Zealand) plasmid miniprep kit. Genomic DNA digests were transferred to positively charged nylon membranes (Roche) by capillary transfer [21] and fixed by UV crosslinking (120,000 μJ/cm^2^) in an Ultraviolet cross-linker Cex-800 (UltraLum, Inc., Claremont, CA 91711, USA). Filters were probed with [α^32^P]-dCTP (3000 Ci/mmol; GE Healthcare, Auckland, New Zealand) labeled probes (Additional File 1). DNA was labeled by primed synthesis with Klenow fragment using a High-Prime kit (Roche). Labeled probes were purified using ProbeQuant™ columns (GE Healthcare). Membranes were washed and hybridization signals detected by autoradiography as described previously [22].

### Gene cloning strategy

Nine SLP genes were identified in *E. festucae *Fl1 either using sequences amplified from the closely related fungal species *N. lolii *Lp19 as described previously [39], or amplified from *E. festucae *Fl1 genomic DNA with degenerate primers (Additional Files 1 and 2). Probes for these genes were hybridized to both an *E. festucae *Fl1 genomic DNA library and a Southern blot containing restriction enzyme digests of *E. festucae *Fl1 genomic DNA. Screening of the genomic library identified clones containing DNA of the gene of interest. Southern hybridizations provided information about the restriction enzyme fragments containing the gene of interest, thus facilitating the subcloning of DNA fragments containing the desired gene. Six further SLP genes were identified in the genome of another *E. festucae *strain, E2368 (Additional file 1).

### Library screening

Construction of the *N. lolii *Lp19 and *E. festucae *Fl1 genomic DNA libraries screened in this study was described previously [23,24]. The *N. lolii *Lp19 genomic DNA library was screened by plaque hybridization using standard methods [25]. For the *E. festucae *Fl1 genomic library prepared as described in [24], filters arrayed with DNA from 5088 independent ampicillin-resistant colonies at a 6 × 6 density with double offset (Australian Genome Research Facility, Melbourne, Australia) were screened by hybridization with radioactively labeled probes [25].

### Polymerase chain reaction and amplification conditions

Standard PCR amplifications of genomic DNA templates were carried out in 25 μL reactions containing 10 mM Tris-HCl, 1.5 mM MgCl_2 _and 50 mM KCl (pH 8.3), 50 μM of each dNTP, 200 nM of each primer, 0.5 U of *Taq *DNA polymerase (Roche) and 5 ng of genomic DNA. The thermocycler conditions used were: 94°C for 2 min; 30 cycles of 94°C for 30 s, 60°C for 30 s and 72°C for 1 min per kb, followed by a final step at 72°C for 5 min.

### DNA sequencing

DNA fragments were sequenced by the dideoxynucleotide chain-termination method [26] using Big Dye (Version 3) chemistry with oligonucleotide primers (Sigma Genosys, Castle Hill, Australia) specific for pUC118, pGEM-T Easy, and genomic sequences from *N. lolii *or *E. festucae*. Products were separated on either an ABI Prism 377 sequencer (Perkin Elmer, Waltham, MA 02451, USA) or an ABI 3730 analyzer (Applied Biosystems, Inc., Foster City, CA 94404, USA) at the Allan Wilson Centre Genome Service, Massey University, Palmerston North, New Zealand.

### Bioinformatic analyses

Sequence data were assembled into contigs with SEQUENCHER (Gene Codes Corporation, Ann Arbor, MI 48108, USA) version 4.1 and analyzed and annotated using MacVector 7.2 (MacVector, Inc., Cary, NC 27519, USA). Sequence comparisons were performed at the National Center of Biotechnology Information (NCBI) site  using the Brookhaven (PDB), SWISSPROT, GenBank (CDS translation), PIR and PRF databases employing algorithms for both local (BLASTX and BLASTP) and global (FASTA) alignments [27-29]. Potential open reading frames for SLP and unlinked non-SLP genes were identified using FGENESH, an HMM-based gene structural prediction using the *Fusarium graminearum *parameters . There were some instances where SLPs were not annotated in genome sequences. TBlastN analysis, using a conserved region of the peptidase S8 domain as the query sequence, was used to identify all putative SLPs in genome sequences. Where additional SLP genes were identified they were included in the analysis. The presence of signal peptides was analyzed using SignalP3.0 [30]. Polypeptide alignments were performed using ClustalW [31] in MEGA4 [32]).

Phylogenetic analyses were conducted in MEGA4 [32]. The evolutionary history was inferred using maximum likelihood (PhyML)[33]. PhyML was run from the ATGC Montpellier Bioinformatics platform at . The Newick files were imported into MEGA 4.0 [32] to view the trees which were saved in tif format. Sequence relationships were inferred using the Neighbor-joining (N-J) method [34]. The bootstrap N-J consensus tree inferred from 1000 replicates was taken to represent the evolutionary history of the taxa analyzed [35]. Branches corresponding to partitions reproduced in less than 50% bootstrap replicates were collapsed. The evolutionary distances were computed using the Poisson correction method [36] and are in the units of the number of amino acid substitutions per site. All positions containing alignment gaps and missing data were eliminated only in pairwise sequence comparisons (Pairwise deletion option).

Assignment of subtilisin-like proteases to different families was done on the basis of domain structure, similarity to other proteases and grouping in phylogenetic trees. Proteinase K type enzymes have propeptide and peptidase S8 domains. Subfamilies sf1, sf2 and sf3 were previously described [4]. Subfamilies sf4 and sf5 of this group were assigned on the basis of their phylogenetic grouping. The pyrolysins have an S8 domain, interrupted by a PA domain, and a DUF1034 domain. The two subfamilies within this group were assigned on the basis of previous work [4]. The OSPs have a peptidase S8 domain and distinct amino acid motifs unique to this family [37].

The following *N. lolii *and *E. festucae *sequences have been submitted to DDBJ/EMBL/Genbank databases: *prtA *and *prtE *(nucleotide accession EU515143/protein accessions ACB30133 and ACB30132), *prtB *(EF015481/ABK27194), *prtC *(FJ648718/ACN30265), *prtD *(EU515141/ACB30128), *prtF *(EU515139/ACB30123), *prtG *(FJ648719/ACN30268), *prtH *(EU515135/ACB30121), *prtI *(EU515134/ACB30118), *prtJ *(FJ648720/ACN30270), *prtK *(EU515134/ACB30119), *prtL *(EU515136/ACB30120), *prtM *(FJ648721/ACN30271), *kexA *(EU515138/ACB30122) and *kexB *(EU515140/ACB30127).

The *E. festucae *strain 2368 genome sequence data are available at .

## Results and discussion

### The *E. festucae *genome contains fifteen members of the subtilisin superfamily

**Figure 1:**
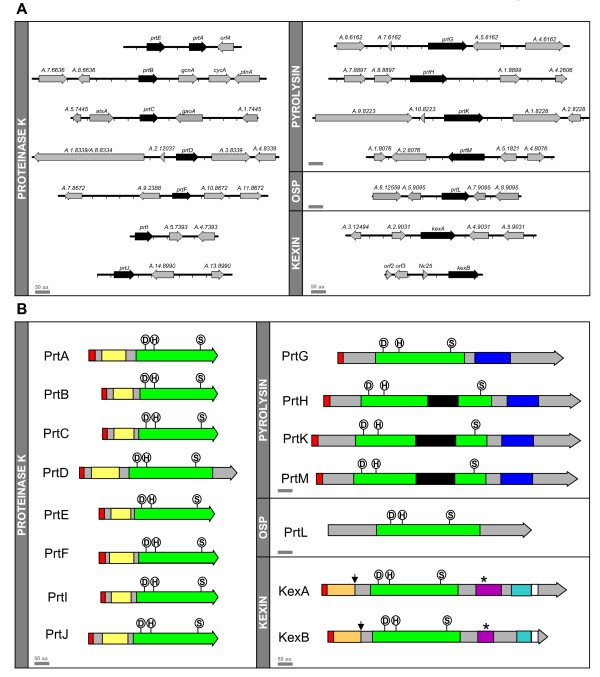
**Gene and protein structures of *E. festucae *Fl1 SLPs**. **A**. Genetic maps showing gene arrangement around the *E. festucae *SLP loci. Black arrows indicate the SLP genes, with non-protease genes indicated by light grey arrows. Gene names, where specific names have been assigned (in *E. festucae *Fl1), are shown above the genes. Systematic numbers assigned during annotation of the *E. festucae *E2368 genome refer to the remaining genes. Scale bars indicate a length of 1 kb. **B**. Domain organization of the *E. festucae *SLP proteins. Conserved catalytic triad residues are shown in circles above domain structures. The polypeptide is indicated in grey and colored blocks as follows: the signal peptide in red, subtilisin N-terminal domains in yellow, peptidase S8 domains in green, PA domains in black, DUF1034 domains in blue, kexin propeptides in orange, P domains in purple, serine/threonine-rich domains in teal and transmembrane domains in white. Conserved kexin autocatalytic cleavage sites are indicated by black arrows. Conserved RGD motifs in the kexin family are indicated by an asterisk. Scale bars indicate a length of 50 amino acid residues.

Using a combination of PCR amplification and whole genome analysis, 15 SLPs were predicted in the genome of the endophytic fungus *E. festucae *(Figure 1A; Additional file 3). *prtA*, *prtE*, *prtB *and *kexB *were initially identified in *N. lolii*, an asexual derivative of *E. festucae *[38]. *prtD*, *prtF*, *prtG *and *prtH *were identified in *E. festucae *strain Fl1 from sequences amplified with degenerate primers designed to an alignment of SLP sequences [39]. During this project, the genome sequence for a closely related strain, *E. festucae *E2368, became available (C. Schardl, B. Roe, U. Hesse and J. W. Jaromczyk, unpublished). *prtI*, *prtJ*, *prtK*, *prtL*, *prtM *and *kexA *were identified from the genome sequence of this *E. festucae *strain (Figure 1A). Direct sequencing of PCR products identified the corresponding genes in *E. festucae *Fl1. The predicted subtilisin-like protease (SLPs) genes (*prtA *and *prtB*) were identified in a genomic library from *N. lolii *[38] (Figure 1A). Probes used to screen the library were amplified with primers based on the *Epichloë typhina At1 *sequence [40]. Another predicted SLP-encoding gene, *prtE*, was identified directly upstream of the *prtA *gene (Figure 1A). Probing a genomic library also identified these three genes in *E. festucae *Fl1. Isolation of the *prtB *gene has been previously described [41]. Further predicted SLP genes were identified in the same library by screening with PCR products amplified using either primers designed to the *E. typhina *At1 gene (*prtC*), or degenerate primers designed to conserved SLP sequences (*prtD, prtF*, *prtG *and *prtH*) (Figure 1A). The *kexB *gene, identified in *N. lolii *directly downstream of the *Nc25 *gene [42], was also identified by probing an *E. festucae *Fl1 genomic library (Figure 1A).

### The *E. festucae *subtilisin superfamily members represent four different families

**Figure 2:**
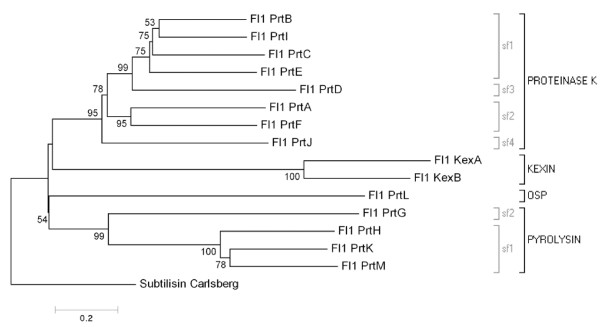
**Evolutionary relationships of *E. festucae *Fl1 SLPs**. Neighbor joining analysis determined phylogenetic relationships. The phylogram is rooted using the *Bacillus subtilis *subtilisin Carlsberg protein (accession P00780) as an outgroup. The percentages of replicate trees in which the associated taxa clustered together in the bootstrap test (1000 replicates) is shown next to the branches. The tree is drawn to scale, with branch lengths in the same units as those of the evolutionary distances used to infer the phylogenetic tree. SLP families and subfamilies are indicated by black or grey square brackets respectively.

Sequence alignments and phylogenetic analysis showed that the predicted *E. festucae *SLPs grouped into four of the six different subtilisin families [3] (Figure 2). Eight genes (*prtA, B, C, D, E, F, I *and *J*) encoded predicted proteins belonging to the proteinase K family (Figure 1B). While none of the *E. festucae *SLPs encoded by these *prt *genes has been tested for protease activity, the PrtC homologue from *E. typhina*, At1, has been shown to have subtilisin-like protease activity [40]. Three subfamilies of proteinase K-like enzymes have been previously identified, two of which are extracellular and one that is vacuolar in localization [4]. The predicted *prtB*, *C*, *E *and *I *gene products belong to subfamily 1, while the predicted *prtA *and *F *gene products belonged to subfamily 2. As expected based on its isolation with degenerate primers designed to members of the vacuolar subfamily, the predicted *prtD *gene product belongs to subfamily 3. Although the predicted *prtJ *gene product was obviously in the proteinase K family, it did not belong to any of the known classes. Instead, it was found in a new subfamily of proteinase K-type enzymes we propose to call subfamily 4 (Figures 2 and 5; Additional file 4). Many other Sordariomycete fungi, as well as some Orbiliomycetes, contain members of this subfamily.

Four genes (*prtG, H, K *and *M*) are predicted to encode proteases from the previously described class I or subtilisin family [4]. In fungi, these proteases contain a peptidase S8 domain, generally interrupted by a protease-associated (PA) domain, sometimes followed by a domain of unknown function (DUF1034) at the carboxyl-terminus. With the exception of PrtG, which is lacking the PA domain insert, the *E. festucae *pyrolysin-like enzymes have this domain structure (Figure 1B). Based on phylogenetic analyses, Hu and St Leger proposed that this family, which they called subtilisin class I, contained two subfamilies of ascomycete proteins [4]. Phylogenetic analysis suggested the predicted *prtH, K *and *M *gene products belonged to subfamily 1, whereas the predicted *prtG *product belonged to subfamily 2 (Figures 2 and 5; Additional file 5).

These gene products may be part of the pyrolysin family, in which proteins have long carboxyl-terminal extensions and large insertions in the catalytic domain [3]. In the basidiomycete wood rot fungus *Pleurotus ostreatus*, a fungal pyrolysin of this type cleaves and activates other proteases, which in turn cleave and activate laccase isoenzymes, in an activation cascade [13]. It remains to be determined if these proteases play similar roles in other fungi.

Both the PA domain and DUF1034 domains have unknown functions. The PA domain may play a role in determining substrate specificity [43], as PA domain insertion in the catalytic peptidase S8 domain may interfere with the substrate reaching the active site [13]. However, in the C5a peptidase (Streptococcus spp.), which has a similar domain structure to the fungal pyrolysins, structural analysis suggests the PA domain is not in a position where it affects substrate specificity [44]. While the function of the DUF1034 domain (PFAM accession PF06280) is unknown, this domain is often present in bacterial and plant SLPs.

The predicted *kexA *and *kexB *gene products belong to the kexin family, which contains enzymes with a specialized role in proprotein processing in the secretory pathway (Figure 5; Additional file 6). The *E. festucae *genome is unusual among ascomycetes in that it contains two kexin-like genes. Like other kexins, the predicted *kexA *and *kexB *gene products both contain putative peptidase S8 and proprotein convertase (P) domains [3] (Figure 1B). The putative P domain in both the predicted KexA and KexB proteins contained an RGD motif, which is conserved in *A. nidulans*, *A. niger *and mammalian furins, but not *S. cerevisiae *Kex2p [45]. The predicted KexA and KexB proteins also contained putative serine/threonine-rich and transmembrane regions downstream of the putative P domain, which are conserved in other kexins. In *S. cerevisiae*, the propeptide of the KEX2 gene product is removed by autocatalysis, with cleavage on the carboxyl-terminal side of a Lys-Arg site [46]. Putative propeptide cleavage sites appeared in both the predicted KexA (Lys112–Arg113) and KexB (Arg112–Arg113) proteins.

The predicted *prtL *gene product did not belong to any of the subtilisin families previously described in fungi. However, sequence comparisons and phylogenetic analysis suggested it was highly similar to a group of proteases called the oxidatively stable proteases (OSPs) [37] (Figures 1B and 5; Additional file 7). The OSPs form a subfamily within the subtilisin family [47]. Like other OSPs, the predicted *prtL *gene product contained many insertions in the peptidase S8 catalytic domain relative to subtilisin Carlsberg, as well as a carboxyl-terminal extension of unknown function that may be required for structural integrity of these enzymes.

### Intron conservation

**Figure 3:**
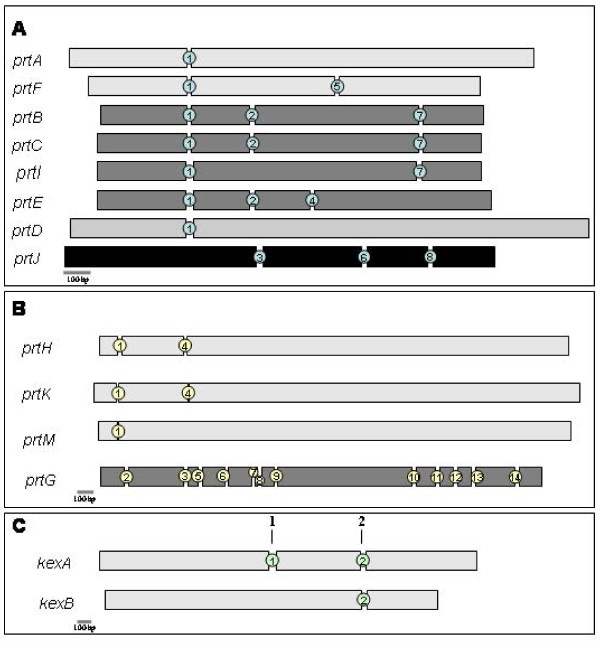
**Exon-intron structures of *E. festucae *SLPs**. Exon-intron structures of the *E. festucae *SLP like genes: proteinase K (A), fungal pyrolysins (B), and kexins (C). Each exon is indicated by a separate rectangle, with spaces between rectangles representing introns. Intron positions found in each family are indicated by numbers between exons. All scale bars indicate a length of 100 bp.

Intron gains and losses are important in the evolution of gene families [48,49]. Intron position was examined in the members of the Fl1 SLP genes (Figure 3). Intron positions were predicted by the gene structure prediction program FGENESH. Sequencing of cDNA amplified from the *prtA*, *prtB*, *prtE *and *kexB *gene products validated the FGENESH predictions for these genes. In the proteinase K family, all of the genes except *prtJ *had a first intron at a conserved position (intron position 1, Figure 3A), suggesting all of these genes were derived from a common ancestral gene. A second intron position was also conserved in *prtB*, *C *and *E *(intron position 2, Figure 3A), while a third intron position was conserved in *prtB*, *C *and *I *(intron position 7, Figure 3A) Comparison of the *prtI *gene with the orthologous *pr1A *gene from *M. anisopliae *revealed that the second of three introns in the *pr1A *gene appears to have been lost from *prtI *(missing intron position 2, Figure 3A). The loss of this intron, and those described subsequently (see below) appears to be due to complete deletion of the intron as there are no apparent relics of the intron left behind. Consequently, the reading frame of the gene is not altered. The *prtF *gene, which is a homologue of the *M. anisopliae pr1J *gene, contained two introns (intron positions 1 and 5, Figure 3A). In the *M. anisopliae *strains where *pr1J *had been sequenced previously, it was suggested an intron was inserted in two strains, rather than the other strain losing an intron [50]. However, the *prtF *gene contains both introns in the same conserved positions as *pr1J*, suggesting that where *pr1J *homologues contained only one intron, this situation has arisen by intron loss. Intron position was not conserved in *prtJ *relative to the other *E. festucae *genes (intron positions 3, 6, and 7, Figure 3A), but was conserved with closely related genes such as FGSG_09382 (*F. graminearum*).

Introns were in conserved positions in three of the four pyrolysin-type genes (Figure 3B). *prtH*, *K *and *M *have a first intron at a conserved position (intron 1, Figure 3B), with a second conserved intron in *prtH *and *prtK*, but apparently lost in *prtM *(intron position 4, Figure 3B). The *prtG *gene did not share any common introns with other Fl1 pyrolysin-type genes (intron positions 2, 3, 5–14; Figure 3B). Both of the kexin type genes, *kexA *and *kexB*, share common introns towards the 3' end of the coding sequence (intron position 2, Figure 3C). However, the *kexA *gene contained an additional intron in the middle of the coding sequence (intron position 1, Figure 3C), which *kexB *did not share. This additional intron is conserved in *Fusarium oxysporum*, *Fusarium verticillioides *and *Trichoderma *spp., but is not found in other fungal species. This suggests that the additional intron has been gained in the Hypocreales lineage. A lack of introns in the *prtL *gene excluded it from this analysis.

### Synteny analysis

**Figure 4:**
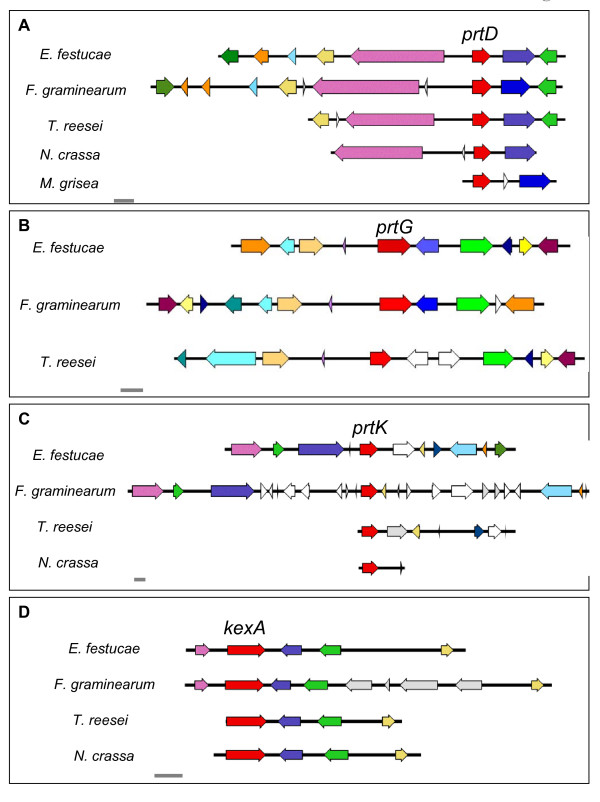
***E. festucae *SLP genes sharing conserved microsynteny with other fungal species**. Conserved microsynteny at the *prtD *(A), *prtG *(B), *prtK *(C). and *kexA *(D) loci. SLP genes are shown in red. Non-orthologous genes are shown in grey or white. Orthologous genes are indicated by colours other than red, grey or white. All scale bars indicate a length of 2 kb.

Sequence analysis revealed that four *E. festucae *Fl1 SLP genes shared microsynteny with the related fungi *Fusarium graminearum*, *Trichoderma reesei*, and in some cases *Neurospora crassa *(Figure 4). These genes were *kexA*, *prtD*, *prtG *and *prtK*. The *kexA *(kexin-like) and *prtD *(vacuolar SLP) genes have highly specialized functions within the cell, which may suggest that conservation of the region around these genes is linked to their conserved function, a hypothesis supported by an analysis of regions of conserved microsynteny between the genomes of *Magnaporthe grisea *and *Neurospora crassa *[51] and other regions of the *E. festucae *genome [24,52]. However, the role of the pyrolysin-like enzymes in fungal cells is not well understood, so it is unclear what significance the synteny of the *prtG *and *prtK *genes may have.

### Comparison of the *E. festucae *SLP family with those from related fungi

In order to determine how the predicted SLP family in *E. festucae *compares with those in other fungal species, a comprehensive survey of these genes in fungal genomes was performed. Numbers of predicted SLP-encoding genes varied from a minimum of three (*Aspergillus nidulans *and *Aspergillus oryzae*), to a maximum of 32 (*Trichoderma virens*) (Additional file 8). The wide variation suggested that the processes of gene duplication and loss have been important in the evolution of this gene family in fungi.

The numbers of predicted SLP genes did not correlate with genome size. For instance, *Aspergillus clavatus *four SLP-encoding genes were inferred in a 35 Mb genome, whereas *Nectria haematococca *had 29 SLP-encoding genes inferred in a 40 Mb genome. Generally, saprotrophic fungi had fewer predicted SLP-encoding genes than the phytopathogenic fungi (Additional file 8); however, a noticeable exception to this trend was the fact that the phytopathogenic *Botrytis cinerea *and *Sclerotinia sclerotiorum *species both had only four SLP genes each, numbers comparable to many of the saprotrophic *Aspergillus *spp.

The types of SLP-encoding genes found in fungal genomes were also classified. Previously, only three classes of SLPs had been identified in fungi: proteinase K, kexin, and a subtilisin-like class. Phylogenetic analyses showed that there appeared to be other SLP classes in fungi. The class first represented by the *M. anisopliae *Pr1C enzyme, described as subtilisin class I [4], contains several unusual features. Unlike most subtilisins, the members of this group often contain an insertion of a protease-associated domain in the subtilisin catalytic domain, and a domain of unknown function, DUF1034, in the carboxyl-terminus. There has been a suggestion that this class of SLPs are pyrolysins [13], which have large insertions and/or carboxyl terminal extensions [3].

### Evolution of the SLP gene family in the Hypocreales

Due to the number of Hypocreales genomes sequenced, this group is a good model to study the evolution of gene families, especially for inferring numbers of ancestral genes and patterns of gene gain and loss. Seven genomes are available within this group: *F. graminearum, F. oxypsorum, F. verticillioides, N. haematococca, T. reesei, Trichoderma virens *and *E. festucae*. Along with data derived from expression studies in *M. anisopliae*, this enabled comparisons to be made between SLP-encoding genes in this group.

**Figure 5:**
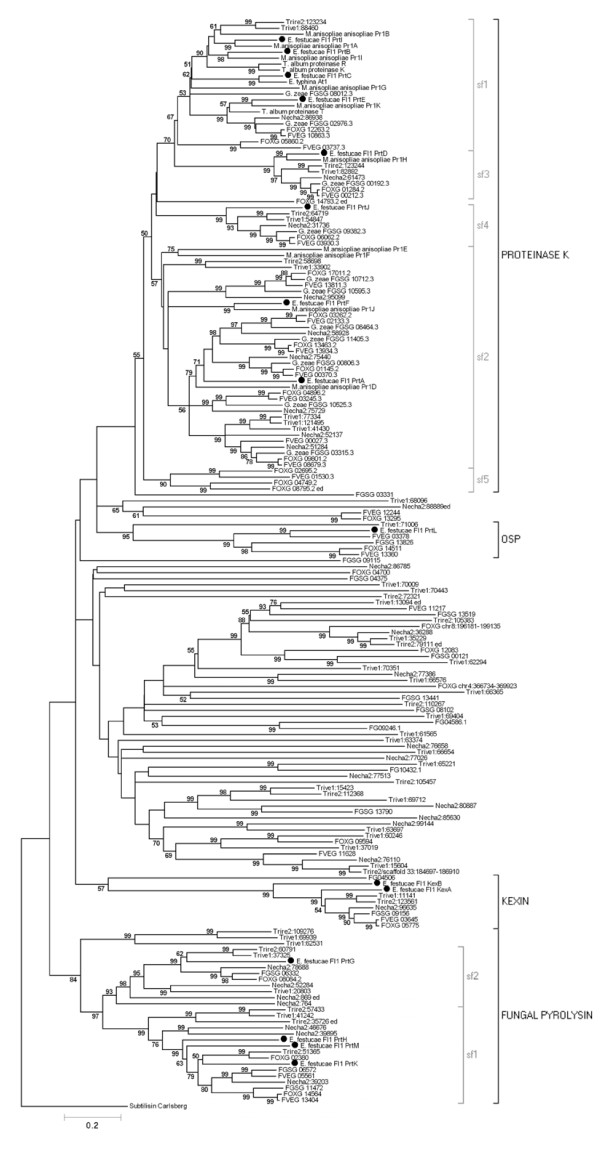
**Evolutionary relationships of Hypocreaceae SLPs based on neighbor joining analysis**. The phylogram is rooted using the *Bacillus subtilis *subtilisin Carlsberg protein (accession P00780) as an outgroup. The percentage of replicate trees in which the associated taxa clustered together in the bootstrap test (1000 replicates) are shown next to the branches. The tree is drawn to scale, with branch lengths in the same units as those of the evolutionary distances used to infer the phylogenetic tree. SLP families and subfamilies are indicated by black or grey square brackets respectively. *E. festucae *sequences are marked by black circles. Note: FOXG_14793 does not group with sf3 (the vacuolar SLPs) or sf4 (characterized by FGSG_09382) but falls into a group by itself. Additional analysis showed it groups with proteases from *P. anserina *and *N. fischeri*.

The first obvious difference between the genome strains was the number of predicted SLP-encoding genes. *E. festucae *had the lowest number of SLP-encoding genes, with just 15 genes present in the genome. This was about half the number found in strains such as *F. graminearum *and *T. virens*. These differences are presumably due to either gene loss in *E. festucae*, or gene duplication in strains with high numbers of SLPs. To test this theory, phylogenetic analysis was used to assess the relationships between the Hypocreales SLPs (Figure 5, Additional file 9).

In the proteinase K family, a complicated pattern of gene duplication and loss has occurred. Analyses suggested that the common ancestor of the Hypocreales must have contained at least six proteinase K type genes, two of which must have belonged to subfamily 1. In *E. festucae *Fl1, the *prtE *and *prtB/prtC/prtI *group represent these genes. For the *prtE *homologues, a gene duplication event seems to have occurred in the *F. graminearum/F. oxysporum/F. verticillioides *lineages, but not in *N. haematococca*. In the case of the *F. oxysporum *and *F. verticillioides *spp. *prtE *homologues, one of the duplicated genes appeared to have lost functionality due to a premature stop codon (FVEG_03737 and FOXG_05860). The *prtE *homologues appear to have been lost from the *Trichoderma *spp. lineages.

The *prtB/prtC/prtI *group was represented in the *Trichoderma *spp. as a single gene, but was lost in the *Fusarium *spp. and *N. haematococca*. The gene ancestral to *prtB/prtC/prtI *appeared to have undergone extensive gene duplication in the Clavicipitaceae lineages (*E. festucae *and *M. anisopliae*) to produce *prtC *(Fl1)/*pr1G *(*M. anisopliae*), *prtB*/*pr1I*, and *prtI*/*pr1A*, along with a further duplication to give the *M. anisopliae pr1B *gene. Pr1A and related enzymes in *M. anisopliae *are thought to act as virulence factors [53], which are effectively in an "arms race" with the protease inhibitors of the insect immune system, so these proteases may have duplicated and diversified to allow the fungi to colonize new hosts. This history of gene duplications is supported by intron position (Figure 3), with the *prtB, prtC, prtE *and *prtI *genes all sharing two common introns, with a third intron shared by *prtB, prtC *and *prtI*.

In proteinase K subfamily 2, there also appears to be three ancestral genes, represented by the *prtF, prtA *and *FGSG_03315 *(*F. graminearum*) genes. *E. festucae *appears to have lost genes from the *FGSG_03315 *group, which is present in one copy in the *T. reesei *and *T. virens *genomes, but in two copies in the *N. haematococca *and *Fusarium *spp. genomes, suggesting that gene duplication has taken place in the common ancestor of these species. In the *prtF *homologues, a single gene is present in all the lineages except the *Trichoderma *spp., where gene loss appears to have occurred, and *F. graminearum*, where a gene duplication event appears to have taken place. The *prtA *homologues may have arisen from duplication of one of the other subfamily 2 genes, as it only contains genes from *Fusarium *spp., *N. haematococca *and the Clavicipitaceae fungi, *E. festucae *and *M. anisopliae*. While the *E. festucae *and *M. anisopliae *genomes contain only a single copy of this gene, two subsequent gene duplications have taken place in the *Fusarium *spp. and *N. haematococca *species to give three *prtA*-like genes for this group.

An unusual case in the *M. anisopliae *genome is the *pr1E *and *pr1F *genes, which are located in tandem. Bagga et al [54] suggest that the ancestor of *M. anisopliae *contained *pr1D *and *pr1J *genes, duplication and divergence of a *pr1J*-like (*prtF *group) gene giving rise to a *pr1F*-likegene, which subsequently reduplicated to give the *pr1E *and *pr1F *genes within the *Metarhizium *genus. The *pr1E *gene appears to have arisen by tandem duplication of the *pr1F *gene within *M. anisopliae*, after divergence of the f. spp. anisopliae and acridum [54].

Proteinase K subfamily 3, containing the specialized vacuolar proteases, is represented by a single gene in all of the Hypocreales strains, suggesting the common ancestor contained this gene. As described earlier, this study revealed the presence of new subfamilies within the proteinase K family. The *prtJ *gene is representative of the new subfamily 4, which is present in a single copy in all of the Hypocreales strains except *M. anisopliae*, where the genome is unsequenced. This gene may be present in the *M. anisopliae *genome, but was undetected during expression studies used to identify SLPs in this organism. The presence of this gene in the Hypocreales genomes (Figure 5) as well as in other Sordariomycete genomes (Additional file 4), suggest this gene was present in the common ancestor.

The newly proposed subfamily 5, characterized by the CHGG_10086 gene from *Chaetomium globosum*, has patchy taxonomic distribution within the Hypocreales, being only found in the *F. oxysporum *and *F. verticillioides *genomes (Figure 5; Additional file 4). This gene appears to have undergone at least one duplication event in the ancestor of these two species to give two genes, followed by another duplication in *F. oxysporum *to give a third gene. However, it is interesting to note that in *F. verticillioides*, frameshifts due to base insertion or deletions have created genes that appear to encode non-functional proteins (FVEG_01530 and FVEG_03386), whereas one of the *F. oxysporum *genes, FOXG_02695, also appears to have undergone a similar frameshift.

Gene duplication and gene loss was studied in the fungal pyrolysin family. For subfamily 2, a single representative was found in each of the Hypocreales genomes, except *M. anisopliae *(possibly due to not having the complete genome sequence). This subfamily was previously shown to have undergone extensive gene duplication in the more distantly related *M. grisea *[4] (Additional file 5). In subfamily 1, gene duplication or loss may have taken place multiple times. All of the Hypocreales genomes contained at least one *prtK*-like gene, with multiple copies in the *Fusarium *spp. and *E. festucae*.

In the kexin family, all Hypocreales strains (except *M. anisopliae*) have at least one kexin gene (Figure 5; Additional file 6). This gene appears to have been duplicated in *E. festucae*. The differences between the sequences appear to indicate divergence of *kexB *from *kexA*.

The *prtL *gene, which represents the OSP subfamily, was present in most of the Hypocreales, except *T. reesei*, where the gene appears to have been lost, and in *F. verticillioides*, where the gene appears to have been duplicated (Figure 5; Additional file 7).

A complicating factor in assessing the evolution of the Hypocreales SLP superfamily is the presence of many sequences with lower SLP similarity in *Trichoderma*, *N. haematococca *and *Fusarium *spp (Figure 5). These sequences, which generally encode large proteins with a peptidase S8 domain characteristic of SLPs, were not present in the *E. festucae *genome, suggesting they may have been lost from these strains. An interesting feature of some of these proteins was the presence of ankyrin repeats in the amino terminus of the protein, with a peptidase S8 domain in the carboxyl terminus (e.g. FGSG_04375 from *F. graminearum*). The role of these proteins within the cell is unknown, but potentially the ankyrin repeats, which are involved in protein-protein interactions [55], could target SLP activity towards particular protein substrates.

## Conclusion

In this study, we aimed to study the evolution of the SLP gene family in *E. festucae*. Fifteen predicted SLP genes were present in the *E. festucae *genome, representing four different SLP families. New subfamilies within the proteinase K family were identified, as well as a new family, the oxidatively stable proteases previously thought to be present only in bacteria. Phylogenetic studies showed that many gene duplications and loss events have occurred during evolution of the SLP gene family within the Hypocreales.

## Authors' contributions

MKB carried out the *E. festucae *Fl1 DNA sequencing, bioinformatics and drafted the manuscript. BS participated in the design and coordination of the study and helped to draft and revise the manuscript. CLS assisted with the phylogenetic analysis and provided the *E. festucae *E2368 sequence. All authors read and approved the final manuscript.

## Supplementary Material

Additional file 1 **Table describing list of probes**. Probes used for Southern hybridization and *E. festucae *genomic library screening. Click here for file

Additional file 2 **Table of primer sequences**. Sequences of primers used to amplify *prt *genes. Click here for file

Additional file 3 **Table showing Bioinformatic analysis of *E. festucae *subtilisin-like protease genes**. Bioinformatic analysis of *E. festucae *strain Fl1 subtilisin-like protease genes. Click here for file

Additional file 4 **Evolutionary relationships of fungal proteinase K family genes based on PhyML analysis**. The phylogram (drawn to scale) is rooted using the *Bacillus subtilis *subtilisin Carlsberg protein (accession P00780) as an outgroup. Numbers at branches indicate the percentage of 1000 bootstrap replicates that supported each branch. *E. festucae *sequences are marked by black circles. Click here for file

Additional file 5 **Evolutionary relationships of fungal pyrolysin genes based on PhyML analysis**. The phylogram (drawn to scale) is rooted using the *Bacillus subtilis *subtilisin Carlsberg protein (accession P00780) as an outgroup. Numbers at branches indicate the percentage of 1000 bootstrap replicates that supported each branch. *E. festucae *sequences are marked by black circles. Click here for file

Additional file 6 **Evolutionary relationships of fungal kexin genes based on PhyML analysis**. The phylogram (drawn to scale) is rooted using the *Bacillus subtilis *subtilisin Carlsberg protein (accession P00780) as an outgroup. Numbers at branches indicate the percentage of 1000 bootstrap replicates that supported each branch. *E. festucae *sequences are marked by black circles. Click here for file

Additional file 7 **Evolutionary relationships of fungal OSP genes based on PhyML analysis**. The phylogram (drawn to scale) is rooted using the *Bacillus subtilis *subtilisin Carlsberg protein (accession P00780) as an outgroup. Numbers at branches indicate the percentage of 1000 bootstrap replicates that supported each branch. *E. festucae *sequences are marked by black circles. Click here for file

Additional file 8 **Table showing taxonomic distribution of subtilisin like proteases**. Taxonomic distribution of subtilisin-like proteases in fungal genomes. Click here for file

Additional file 9 **Table on distribution of subtilisin like proteases**. Distribution of Hypocreales subtilisin-like proteases in known families and subfamilies. Click here for file
