# Supplementary material for: Evolution of a subtilisin-like protease gene family in the grass endophytic fungus Epichloë festucae
Source: BMC Evol Biol. 2009 Jul 19;9:168. doi: 10.1186/1471-2148-9-168 (PMC2717940; doi:10.1186/1471-2148-9-168)
Supplement: Additional file 1 — Table describing list of probes. Probes used for Southern hybridization and E. festucae genomic library screening. [file 1471-2148-9-168-S1.doc]

**Additional File 1**: Probes used for Southern hybridization and *E. festucae* genomic library screening.

| Gene | Sequence Source | Probe | Reference |
| --- | --- | --- | --- |
| *prtA* | *N. lolii* strain Lp19 | MM5-MM2 | [38, 39] |
| *prtB* | *N. lolii* strain Lp19 | MM15-MM6 | [38, 39] |
| *prtC* | *N. lolii* strain Lp19 | MM75-MM76 | [38] |
| *prtD^a^* | PCR of *E. festucae* strain Fl1 genomic DNA | MM93-MM94 | [38] |
| *prtE* | *N. lolii* strain Lp19 | MM155-MM130 | [38] |
| *prtF^b^* | PCR of *E. festucae* strain Fl1 genomic DNA | MM149-MM150 | [38] |
| *prtG^b^* | PCR of *E. festucae* strain Fl1 genomic DNA | MM149-MM150 | [38] |
| *prtH^b^* | PCR of *E. festucae* strain Fl1 genomic DNA | MM149-MM150 | [38] |
| *prtI* | *E. festucae* strain E2368 genome |  | This study |
| *prtJ* | *E. festucae* strain E2368 genome |  | This study |
| *prtK* | *E. festucae* strain E2368 genome |  | This study |
| *prtL* | *E. festucae* strain E2368 genome |  | This study |
| *prtM* | *E. festucae* strain E2368 genome |  | This study |
| *kexA* | *E. festucae* strain E2368 genome |  | This study |
| *kexB* | *N. lolii* strain Lp19 | MM141-MM142 | This study |

^a^Degenerate primers were designed to conserved regions identified in alignments of vacuolar subtilisin-like proteases [39].

^b^Degenerate primers were designed to conserved regions identified in alignments of all available subtilisin-like proteases [39].
