# Supplementary material for: Evolution of a subtilisin-like protease gene family in the grass endophytic fungus Epichloë festucae
Source: BMC Evol Biol. 2009 Jul 19;9:168. doi: 10.1186/1471-2148-9-168 (PMC2717940; doi:10.1186/1471-2148-9-168)
Supplement: Additional file 2 — Table of primer sequences. Sequences of primers used to amplify prt genes. [file 1471-2148-9-168-S2.doc]

**Additional File 2:** Sequences of primers used to amplify *prt* genes

| Primer name | Sequence (5’ to 3’) | Gene |
| --- | --- | --- |
| MM2 | GTGATCCAGTCGAGAGTC | *prtA* |
| MM5 | TGATGCCTGGACATGTTG | *prtA* |
| MM15 | GTGACATTGGTGGCTACG | *prtB* |
| MM6 | TCGTTCAGCGACTGCGAG | *prtB* |
| MM75 | GCCTCGAACGAGTACGTC | *prtC* |
| MM76 | TGGTTTGTGGCCTTGGAG | *prtC* |
| MM93 | GATCAYGTAGATTTYGARGG | *prtD* |
| MM94 | GCATCAGCGTTATCRTTNCC | *prtD* |
| MM155 | TACAGCCACTCCTTCAAC | *prtE* |
| MM130 | TAGTCCATGCCAGCAATG | *prtE* |
| MM149^a^ | GGNCAYGGNACNCAYGTNGC | *prtF, prtG, prtH* |
| MM150^a^ | GGNSWNGCCATNGANGTNCC | *prtF, prtG, prtH* |
| MM141 | ATGTACAGCCACGATTGG | *kexB* |
| MM142 | CATAGTAGTAAGACAACG | *kexB* |

^a^These degenerate primers amplified several different products, three of which contained *prt* gene sequences designated as *prtF*, *prtG* and *prtH,* respectively.
