# Supplementary material for: Evolution of a subtilisin-like protease gene family in the grass endophytic fungus Epichloë festucae
Source: BMC Evol Biol. 2009 Jul 19;9:168. doi: 10.1186/1471-2148-9-168 (PMC2717940; doi:10.1186/1471-2148-9-168)
Supplement: Additional file 3 — Table showing Bioinformatic analysis of E. festucae subtilisin-like protease genes. Bioinformatic analysis of E. festucae strain Fl1 subtilisin-like protease genes. [file 1471-2148-9-168-S3.doc]

| Gene | Nucleotide accession | Protein accession | Secreted^a^ | Preproprotein^b^ | Predicted introns | Best hit^c^ | Family | Subfamily | *M. anisopliae* match | *G. zeae* match | Synteny with *G. zeae* |
| --- | --- | --- | --- | --- | --- | --- | --- | --- | --- | --- | --- |
| *prtA* | EU515143 | ACB30132 | Y | 434 aa | 1 | FG00806 *Gibberella zeae* | proteinase K | 2 |  | FG00806 |  |
| *prtB* | EF015481 | ABK27194 | Y | 389 aa | 3 | Pr1I *Metarhizium anisopliae* | proteinase K | 1 | Pr1I |  |  |
| *prtC* | FJ648718 | ACN30265 | Y | 388 aa | 3 | At1 *Epichloë typhina* | proteinase K | 1 | Pr1G |  |  |
| *prtD* | EU515141 | ACB30128 | Y | 531 aa | 1 | Pr1H *M. anisopliae* | proteinase K | 3 | Pr1H | FG00192 | √ |
| *prtE* | EU515143 | ACB30133 | Y | 395 aa | 3 | Pr1K *M. anisopliae* | proteinase K | 1 | Pr1K | FG02976 |  |
| *prtF* | EU515139 | ACB30123 | Y | 401 aa | 2 | Pr1J *M. anisopliae* | proteinase K | 2 | Pr1J |  |  |
| *prtG* | FJ648719 | ACN30268 | Y | 761 aa | 11 | FG06332 *G. zeae* | pyrolysin | 2 |  | FG06332 | √ |
| *prtH* | EU515137 | ACB30121 | Y | 866 aa | 1 | Pr1C *M. anisopliae* | pyrolysin | 1 | Pr1C |  |  |
| *prtI* | EU515134 | ACB30118 | Y | 389 aa | 2 | Pr1A *M. anisopliae* | proteinase K | 1 | Pr1A |  |  |
| *prtJ* | FJ648720 | ACN30270 | Y |  | 3 | FG09382 *G. zeae* | proteinase K | 4 |  | FG09382 |  |
| *prtK* | EU515135 | ACB30119 | Y | 906 aa | 2 | Pr1C *M. anisopliae* | pyrolysin | 1 | Pr1C | FG06572 | √ |
| *prtL* | EU515136 | ACB30120 | N | 696 aa | 0 | Arth_3479 *Arthrobacter* sp. FB24 | true subtilisin | OSP |  | FG11223 |  |
| *prtM* | FJ648721 | ACN30271 | Y | 880 aa | 2 | Pr1C *M. anisopliae* | pyrolysin | 1 | Pr1C |  |  |
| *kexA* | EU515138 | ACB30122 | Y | 825 aa | 2 | FG09156 *G. zeae* | kexin |  |  | FG09156 | √ |
| *kexB* | EU515140 | ACB30127 | Y | 742 aa | 1 | An01g08530 *Aspergillus niger* | kexin |  |  |  |  |

^a^ Presence of signal peptide was determined by SignalP 3.0 analysis.

^b^ Length of protein before post-translational protein processing is shown.

^c^ Best hit defined as closest match identified by BlastP analysis
