# Supplementary figures and images for: Evolution of a subtilisin-like protease gene family in the grass endophytic fungus Epichloë festucae
Source: BMC Evol Biol. 2009 Jul 19;9:168. doi: 10.1186/1471-2148-9-168 (PMC2717940; doi:10.1186/1471-2148-9-168)

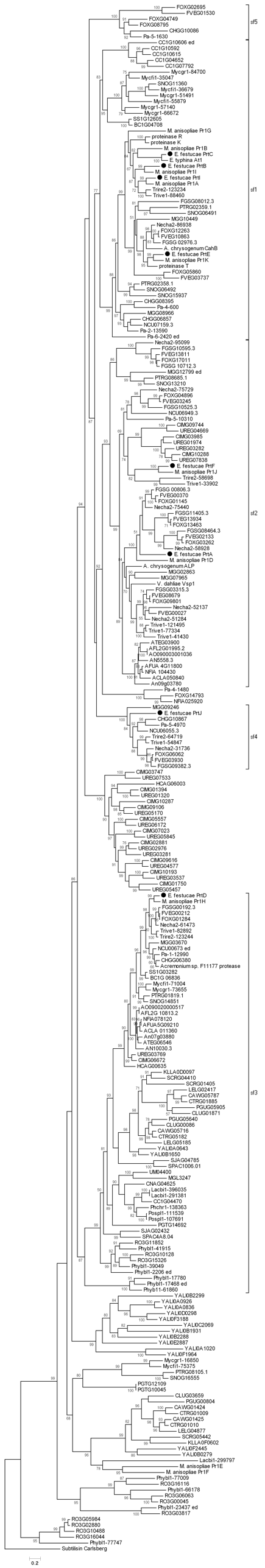

Supplement: Additional file 4 — Evolutionary relationships of fungal proteinase K family genes based on PhyML analysis. The phylogram (drawn to scale) is rooted using the Bacillus subtilis subtilisin Carlsberg protein (accession P00780) as an outgroup. Numbers at branches indicate the percentage of 1000 bootstrap replicates that supported each branch. E. festucae sequences are marked by black circles. [file 1471-2148-9-168-S4.jpeg]

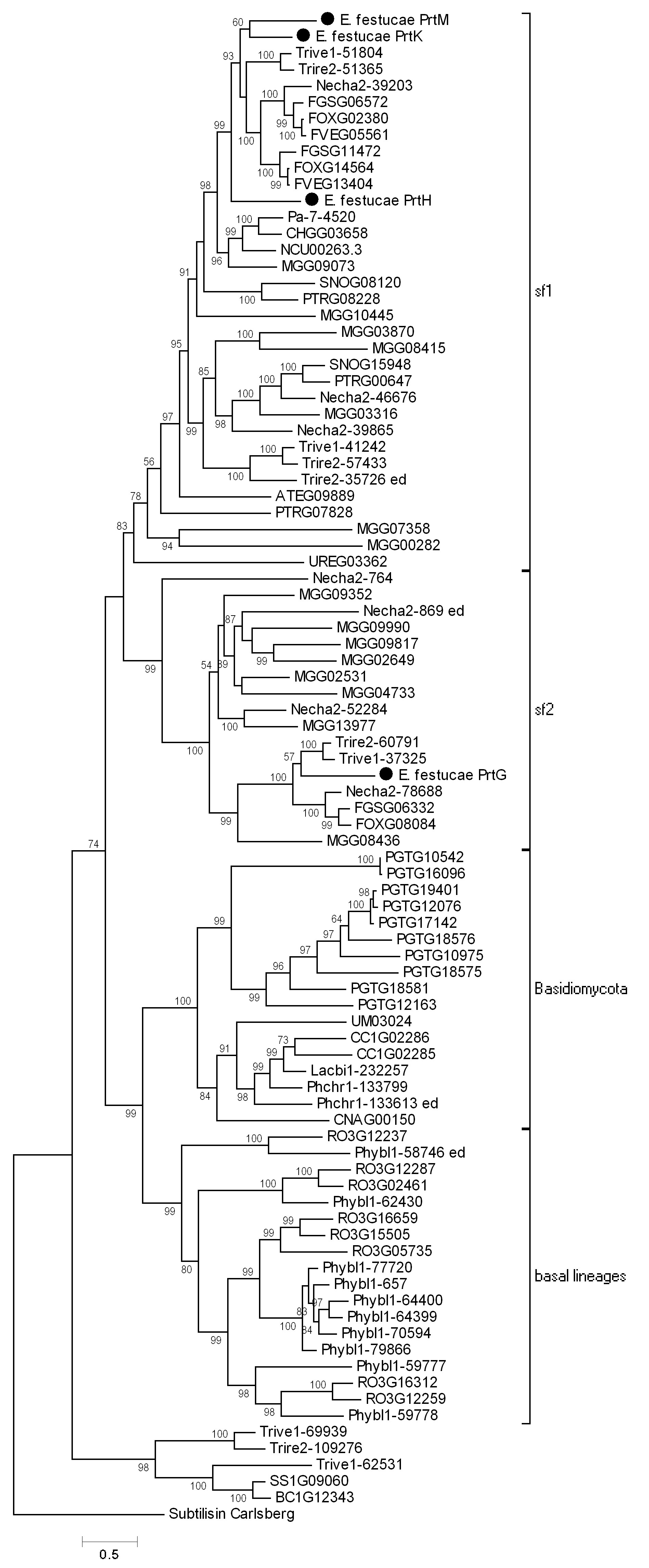

Supplement: Additional file 5 — Evolutionary relationships of fungal pyrolysin genes based on PhyML analysis. The phylogram (drawn to scale) is rooted using the Bacillus subtilis subtilisin Carlsberg protein (accession P00780) as an outgroup. Numbers at branches indicate the percentage of 1000 bootstrap replicates that supported each branch. E. festucae sequences are marked by black circles. [file 1471-2148-9-168-S5.jpeg]

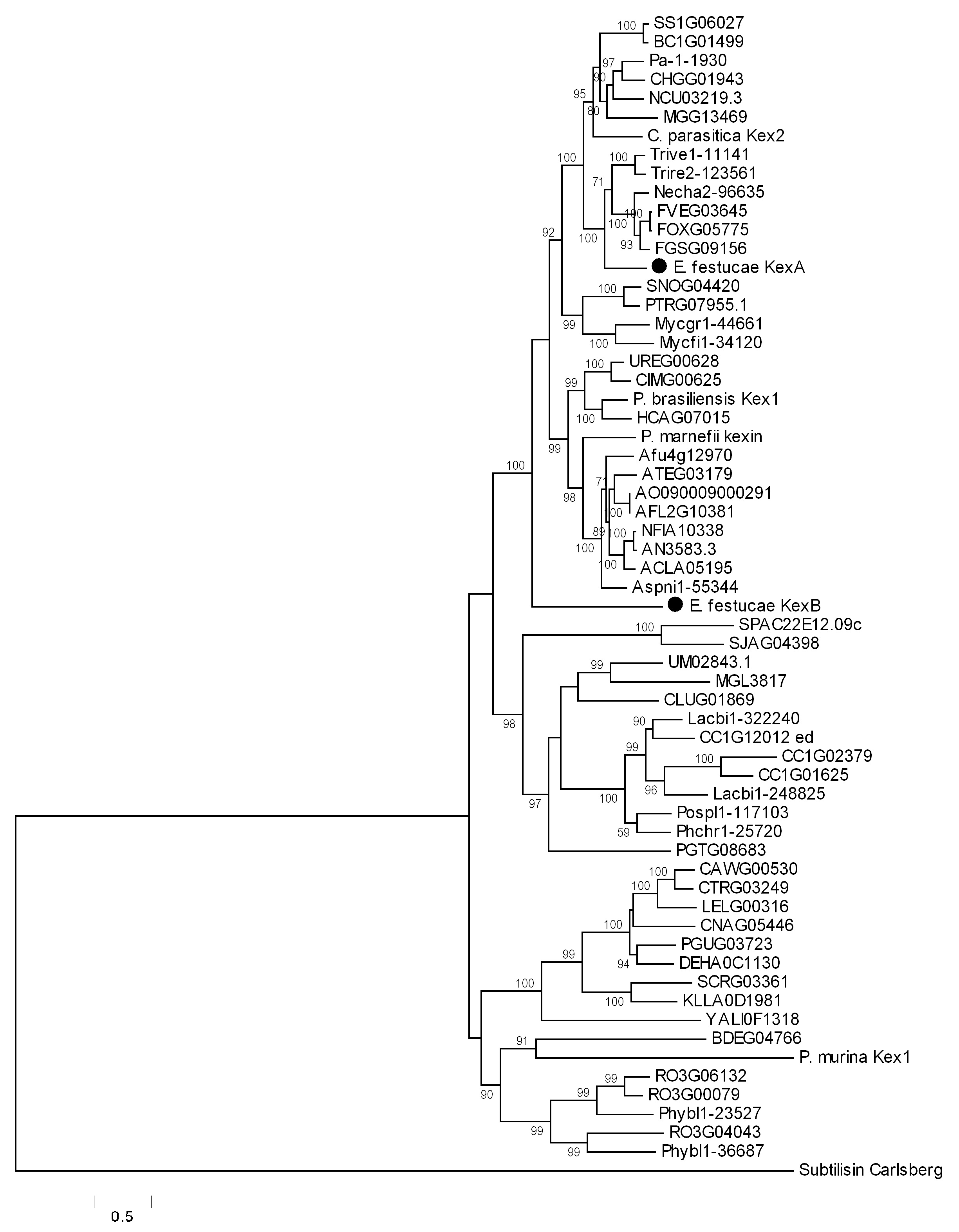

Supplement: Additional file 6 — Evolutionary relationships of fungal kexin genes based on PhyML analysis. The phylogram (drawn to scale) is rooted using the Bacillus subtilis subtilisin Carlsberg protein (accession P00780) as an outgroup. Numbers at branches indicate the percentage of 1000 bootstrap replicates that supported each branch. E. festucae sequences are marked by black circles. [file 1471-2148-9-168-S6.jpeg]

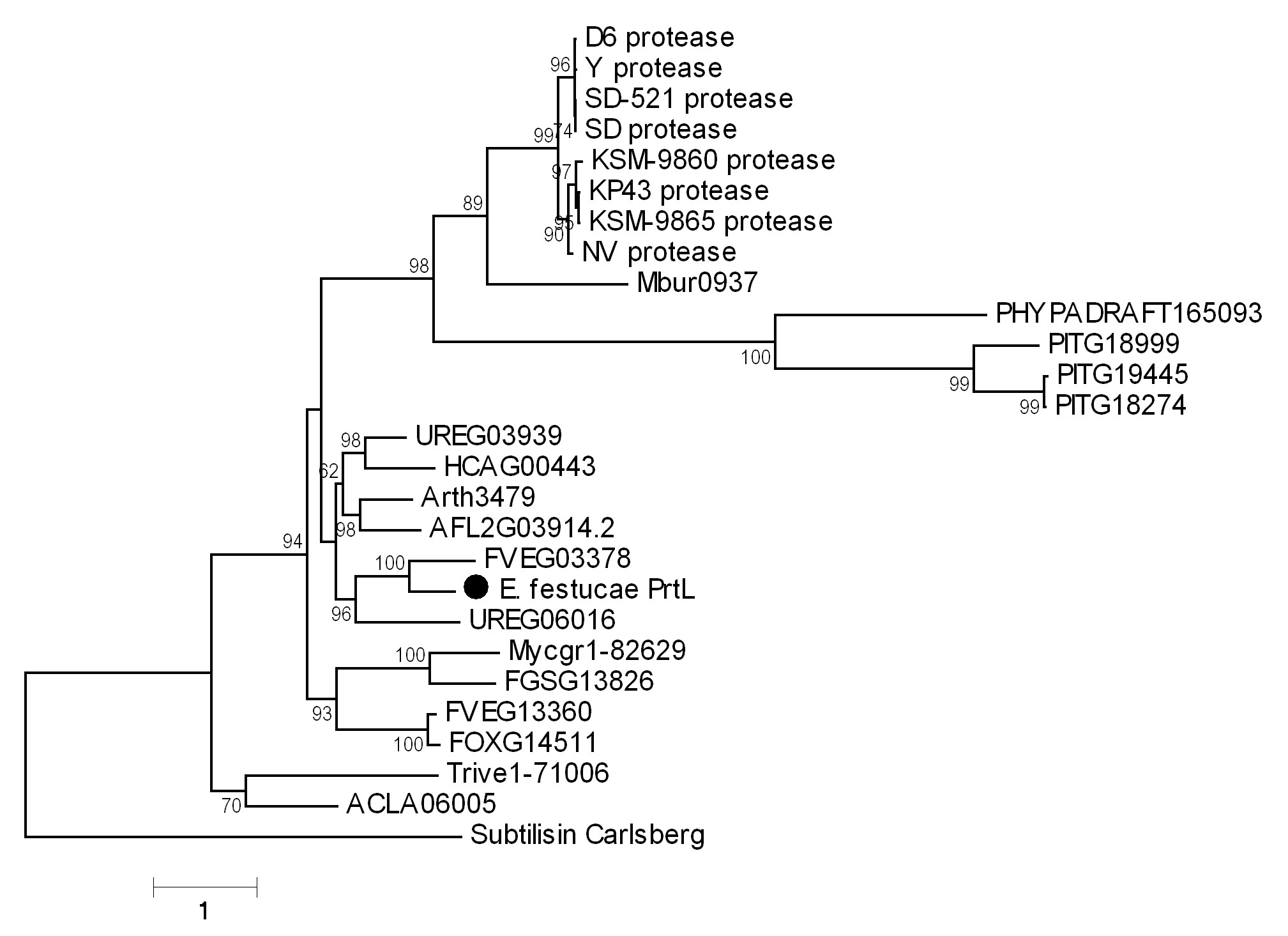

Supplement: Additional file 7 — Evolutionary relationships of fungal OSP genes based on PhyML analysis. The phylogram (drawn to scale) is rooted using the Bacillus subtilis subtilisin Carlsberg protein (accession P00780) as an outgroup. Numbers at branches indicate the percentage of 1000 bootstrap replicates that supported each branch. E. festucae sequences are marked by black circles. [file 1471-2148-9-168-S7.jpeg]
