# Supplementary material for: Evolution of a subtilisin-like protease gene family in the grass endophytic fungus Epichloë festucae
Source: BMC Evol Biol. 2009 Jul 19;9:168. doi: 10.1186/1471-2148-9-168 (PMC2717940; doi:10.1186/1471-2148-9-168)
Supplement: Additional file 8 — Table showing taxonomic distribution of subtilisin like proteases. Taxonomic distribution of subtilisin-like proteases in fungal genomes. [file 1471-2148-9-168-S8.doc]

| PHYLUM | SUBPHYLUM | CLASS | ORDER | ORGANISM | LIFESTYLE | NOTES | GENOME SIZE | CS #^a^ | PYRO | PROT K | | KEXIN | OSP | OTHER | TOTAL |
| --- | --- | --- | --- | --- | --- | --- | --- | --- | --- | --- | --- | --- | --- | --- | --- |
|  |  |  |  |  |  |  |  |  |  | NON VAC | VAC |  |  |  |  |
| ASCOMYCOTA | PEZIZOMYCOTINA | DOTHIDEOMYCETES | CAPNODIALES | *Mycosphaerella fijiensis*^g^ | phytopathogen | hemibiotroph | 70 Mb |  | 0 | 4 | 1 | 1 | 0 | 1 | 7 |
|  |  |  |  | *Mycosphaerella graminicola*^g^ | phytopathogen | hemibiotroph | 31 Mb |  | 0 | 6 | 1 | 1 | 1 | 1 | 10 |
|  |  |  | PLEOSPORALES | *Pyrenophora tritici-repentis*^f^ | phytopathogen | necrotrophic | 37 Mb |  | 3 | 3 | 2 | 1 | 0 | 0 | 9 |
|  |  |  |  | *Stagonospora nodorum*^f^ | phytopathogen | necrotrophic | 37 Mb |  | 2 | 6 | 1 | 1 | 0 | 0 | 10 |
|  |  | EUROTIOMYCETES | EUROTIALES | *Aspergillus clavatus NRRL-1*^f^ | saprotroph | rarely pathogenic | 35 Mb |  | 0 | 1 | 1 | 1 | 1 | 0 | 4 |
|  |  |  |  | *Aspergillus flavus*^f^ | saprotroph | pathogenic | 36 Mb | 8 | 0 | 1 | 1 | 1 | 1 | 1 | 5 |
|  |  |  |  | *Aspergillus fumigatus*^f^ | saprotroph | opportunistic pathogen | 30 Mb |  | 0 | 3 | 1 | 1 | 0 | 0 | 5 |
|  |  |  |  | *Aspergillus nidulans*^f^ | saprotroph | model organism | 31 Mb | 8 | 0 | 1 | 1 | 1 | 0 | 0 | 3 |
|  |  |  |  | *Aspergillus niger*^g^ | saprotroph | important industrially | 37 Mb |  | 0 | 1 | 1 | 1 | 0 | 2 | 5 |
|  |  |  |  | *Aspergillus oryzae*^f^ | saprotroph | found soil & plant parts | 37 Mb |  | 0 | 1 | 1 | 1 | 0 | 0 | 3 |
|  |  |  |  | *Aspergillus terreus*^f^ | saprotroph | can be pathogenic | 35 Mb | 26 | 1 | 1 | 1 | 1 | 0 | 2 | 6 |
|  |  |  |  | *Neosartorya fischeri NRRL 181*^f^ | saprotroph | pathogenic | 35 Mb |  | 0 | 4 | 1 | 1 | 0 | 0 | 6 |
|  |  |  | ONYGENALES | *Coccidioides immitis RS*^f^ | saprotroph | pathogenic (valley fever) | 29 Mb |  | 0 | 13 | 1 | 1 | pseudo | 1 | 16 |
|  |  |  |  | *Histoplasma capsulatum*^f^ | pathogen | causes histoplasmosis | 24 Mb |  | 0 | 1 | 1 | 1 | 1 | 1 | 5 |
|  |  |  |  | *Uncinocarpus reesei*^f^ | saprotroph | nonpathogenic | 30 Mb |  | 1 | 14 | 1 | 1 | 2 | 0 | 19 |
|  |  | LEOTIOMYCETES | HELOTIALES | *Botrytis cinerea*^f^ | phytopathogen | necrotrophic | 38 Mb |  | 1 | 1 | 1 | 1 | 0 | 0 | 4 |
|  |  |  |  | *Sclerotinia sclerotiorum*^f^ | phytopathogen | necrotrophic | 38 Mb |  | 1 | 1 | 1 | 1 | 0 | 0 | 4 |
|  |  | SORDARIOMYCETES | HYPOCREALES | *Epichloë festucae*^h^ | symbiont | mutualistic symbiont | 29 Mb |  | 4 | 7 | 1 | 2 | 1 | 0 | 15 |
|  |  |  |  | *Fusarium graminearum*^f^ | phytopathogen | biotrophic | 40 Mb | 4 | 3 | 10 | 1 | 1 | 1 | 13 | 29 |
|  |  |  |  | *Fusarium oxysporum*^f^ | phytopathogen | biotrophic | 60 Mb |  | 3 | 13 | 1 | 1 | 1 | 7 | 26 |
|  |  |  |  | *Fusarium verticillioides*^f^ | phytopathogen | biotrophic | 46 Mb | 12 | 2 | 12 | 1 | 1 | 1 | 4 | 21 |
|  |  |  |  | *Nectria haematococca MP VI*^g^ | phytopathogen | biotrophic | 40 Mb |  | 7 | 8 | 1 | 1 | 0 | 12 | 29 |
|  |  |  |  | *Trichoderma reesei*^g^ | mycopathogen |  | 33 Mb | 7 | 5 | 4 | 1 | 1 | 0 | 7 | 18 |
|  |  |  |  | *Trichoderma virens*^g^ | mycopathogen |  |  |  | 4 | 5 | 1 | 1 | 1 | 20 | 32 |
|  |  |  | S INCERTAESEDIS^b^ | *Magnaporthe grisea*^f^ | phytopathogen | rice blast | 40 Mb | 7 | 16 | 6 | 1 | 1 | 0 | 0 | 24 |
|  |  |  | SORDIALES | *Chaetomium globosum*^f^ | saprotroph | skin & nail infections | 36 Mb |  | 1 | 4 | 1 | 1 | 0 | 2 | 9 |
|  |  |  |  | *Neurospora crassa*^f^ | saprotroph |  | 43 Mb | 7 | 1 | 3 | 1 | 1 | 0 | 1 | 7 |
|  |  |  |  | *Podospora anserina*^i^ | saprotroph |  | 34 Mb | 7 | 1 | 7 | 1 | 1 | 0 | 0 | 10 |
|  | SACCHAROMYCOTINA | SACCHAROMYCETES | SACCHAROMYCETALES | *Candida albicans*^f^ | pathogen | opportunistic pathogen | 16 Mb |  | 0 | 3 | 1 | 1 | 0 | 0 | 5 |
|  |  |  |  | *Candida guilliermondii*^f^ | pathogen | opportunistic pathogen | 12 Mb |  | 0 | 2 | 1 | 1 | 0 | 0 | 4 |
|  |  |  |  | *Candida lusitaniae*^f^ | pathogen | opportunistic pathogen | 16 Mb |  | 0 | 2 | 1 | 1 | 0 | 0 | 4 |
|  |  |  |  | *Candida tropicalis*^f^ | pathogen | opportunistic pathogen | 15 Mb |  | 0 | 2 | 2 | 1 | 0 | 0 | 5 |
|  |  |  |  | *Debaryomyces hansenii*^f^ | saprotroph |  | 12 Mb | 7 | 0 | 2 | 1 | 1 | 0 | 0 | 4 |
|  |  |  |  | *K. lactis NRRL Y-1140*^cf^ | saprotroph |  | 10 Mb | 6 | 0 | 2 | 1 | 1 | 0 | 0 | 4 |
|  |  |  |  | *Lodderomyces elongisporus*^f^ | saprotroph? | nonpathogenic | 16 Mb |  | 0 | 2 | 1 | 1 | 0 | 0 | 4 |
|  |  |  |  | *S. cerevisiae Rlm-11*^df^ | saprotroph | natural isolate | 12 Mb | 16 | 0 | 2 | 1 | 1 | 0 | 0 | 4 |
|  |  |  |  | *Yarrowia lipolytica*^j^ | saprotroph |  | 20 Mb | 6 | 0 | 1 | 2 | 1 | 0 | 0 | 4 |
|  | SCHIZOSACCHAROMYCOTINA | SCHIZOSACCHAROMYCETES | SCHIZOSACCHAROMYCETALES | *S japonicus*^ef^ | saprotroph | dimorphic | 14 Mb |  | 0 | 1 | 1 | 1 | 0 | 1 | 4 |
|  |  |  |  | *Schizosaccharomyces pombe*^f^ | saprotroph | model organism | 13 Mb | 3 | 0 | 1 | 1 | 1 | 0 | 1 | 4 |
| BASIDIOMYCOTA | AGARICOMYCOTINA | AGARICOMYCETES | AGARICALES | *Coprinus cinereus*^f^ | mushroom |  | 37 Mb | 13 | 2 | 6 | 1 | 3 | 0 | 0 | 12 |
|  |  |  |  | *Laccaria bicolor*^g^ | symbiont | symbiotic: ectomycorrhizal | 61 Mb |  | 1 | 2 | 1 | 2 | 0 | 0 | 6 |
|  |  |  | CORITCALES | *Phanerochaete chrysosporium*^g^ | saprotroph | degrades wood | 30 Mb | 10 | 2 | 0 | 1 | 1 | 0 | 0 | 4 |
|  |  | TREMELLOMYCETES | TREMELLATALES | *Cryptococcus neoformans ser.*^f^ *A* | pathogenic |  | 20 Mb | 14 | 1 | 0 | 1 | 1 | 0 | 0 | 3 |
|  | PUCCINOMYCOTINA | PUCCINOMYCETES | PUCCINALES | *Puccinia graminis*^f^ | phytopathogen | obligate | 81 Mb | 18 | 10 | 2 | 1 | 1 | 0 | 0 | 14 |
|  | USTILAGINOMYCOTINA | USTILAGINOMYCETES | USTILAGINALES | *Ustilago maydis*^f^ | phytopathogen | biotrophic | 20 Mb | 23 | 1 | 0 | 1 | 1 | 0 | 1 | 4 |
| BASAL | MUCORMYCOTINA |  | MUCORALES | *Phycomyces blakesleeanus*^g^ | saprotroph |  | 40 Mb |  | 10 | 8 | 3 | 2 | 0 | 1 | 24 |
|  |  |  |  | *Rhizopus oryzae*^f^ | saprotroph |  | 40 Mb |  | 8 | 8 | 3 | 3 | 0 | 1 | 23 |

^a^ The number of chromosomes is shown only if information was available.

^b^Sordariomycetes incertae sedis

^c^Kluveromyces lactis

^d^Saccharomyces cerevisiae

^e^Schizosaccharomyces japonicus

^f^http://www.broad.mi.edu/data – genome data source

^g^http://www.jgi.doe.gov – genome data source

^h^http://www.genome.ou.edu/fungi.html - genome data source

^i^http://www.podospora.igmors.u-psud.fr - genome data source

^j^http://www.ncbi.nlm.nih.gov - genome data source
