# Supplementary material for: Evolution of a subtilisin-like protease gene family in the grass endophytic fungus Epichloë festucae
Source: BMC Evol Biol. 2009 Jul 19;9:168. doi: 10.1186/1471-2148-9-168 (PMC2717940; doi:10.1186/1471-2148-9-168)
Supplement: Additional file 9 — Table on distribution of subtilisin like proteases. Distribution of Hypocreales subtilisin-like proteases in known families and subfamilies. [file 1471-2148-9-168-S9.doc]

|  | **PROTEINASE K** | | | | | | | | | | | | | | |
| --- | --- | --- | --- | --- | --- | --- | --- | --- | --- | --- | --- | --- | --- | --- | --- |
|  | subfamily 1 | | | | | | subfamily 2 | | | | | | | | subfamily 3 |
|  | *prtE* group | | *prtB/C/I* group | | | | *prtA* group | | | *prtF* group | | FGSG_03315 group | | |  |
| *E. festucae* | *prtE* | | *prtB* | *prtC* | *prtI* | *-* | *prtA* | | | *prtF* | | *-* | | | *prtD* |
| *M. anisopliae* | *pr1K* | | *pr1I* | *pr1G* | *pr1A* | *pr1B* | *pr1D* | | | *pr1J* | *pr1E/pr1F* | b | | | *pr1H* |
| *F. oxysporum* | FOXG_12263 | FOXG_05680^a^ | - | | | | FOXG_01145 | FOXG_03262 | FOXG_13463 | FOXG_17011 | | FOXG_04896 | FOXG_09801 | | FOXG_01284 |
| *F. verticillioides* | FVEG_10863 | FVEG_03737^a^ | - | | | | FVEG_00370 | FVEG_02133 | FVEG_13934 | FVEG_13811 | | FVEG_03245 | FVEG_08679 | | FVEG_00212 |
| *G. zeae* | FGSG_02976 | FGSG_08012 | - | | | | FGSG_00806 | FGSG_08464 | FGSG_11405 | FGSG_10595 | FGSG_10712 | FGSG_10525 | FGSG_03315 | | FGSG_00192 |
| *N. haematococca* | Necha:86938 | | - | | | | Necha2:75440 | Necha2:58928 | | Necha2:95099 | | Necha2:75729 | Necha2:52137 | Necha2:51284 | Necha2:61473 |
| *T. reesei* | - | | Trire2:123234 | | | | Trire2:58698 | | | Trire2:121495 | | - | | | Trire2:123244 |
| *T. virens* | - | | Trive1:88460 | | | | Trive1:33902 | | | Trive1:41430 | | Trive1:77344 | | | Trive1:82892 |
|  | **PYROLYSIN** | | | | | | | | | **KEXIN** | | | **OSP** | | |
|  | subfamily 1 | | subfamily 2 | | | | other | | |  |  |  |  |  |  |
| *E. festucae* | *prtK* | *prtH/prtM* | *prtG* | | | |  | | | *kexA* | *kexB* | | *prtL* | | |
| *M. anisopliae* | pr1C^b^ | | b | | | |  | | | b | | | b | | |
| *F. oxysporum* | FOXG_02380 | FOXG_14564 | FOXG_08084 | | | |  | | | FOXG_05775 | | | FOXG_14511 | | |
| *F. verticillioides* | FVEG_05561 | FVEG_13404 | - | | | |  | | | FVEG_03645 | | | FVEG_03378 | | FVEG_13660 |
| *G. zeae* | FGSG_06572 | FGSG_11472 | FGSG_06332 | | | |  | | | FGSG_09156 | | | FGSG_13826 | | |
| *N. haematococca* | Necha2:39203 | Necha2:46676/Necha2:39895 | Necha2:78688 | | | |  | | | Necha2:96635 | | |  | | |
| *T. reesei* | Trire2:51365 | Trire2:57433/Trire2:35726 | Trire2:60791 | | | | Trire2:109276 | | | Trire2:123561 | | |  | | |
| *T. virens* | - | Trive1:41242 | Trive1:37325 | | | | Trive1:69939 | | Trive1:62531 | Trive1:11141 | | | Trive1:71006 | | |

^a^ These genes may be pseudogenes due to presence of apparent insertions or deletions that cause frameshifts and premature truncation of the polypeptide product.

^b^ More genes from these families may be present in this species, but have not yet been identified.
